# Herbal Medicine from Single Clove Garlic Oil Extract Ameliorates Hepatic Steatosis and Oxidative Status in High Fat Diet Mice

**DOI:** 10.21315/mjms2020.27.1.5

**Published:** 2020-02-27

**Authors:** Siti Nur Arifah, Mochammad Fitri Atho’illah, Betty Lukiati, Sri Rahayu Lestari

**Affiliations:** 1 Department of Biology, Faculty of Mathematics and Natural Sciences, Universitas Negeri Malang, East Java, Indonesia; 2 Department of Biology, Faculty of Mathematics and Natural Sciences, Brawijaya University, East Java, Indonesia

**Keywords:** single clove garlic oil, superoxide dismutase, tumour necrosis factor-α, hepaticsteatosis, high fat diet

## Abstract

**Introduction:**

High fat diet (HFD) can cause lipid accumulation and contribute to various metabolic disorders. Single clove garlic oil (SCGO) has advantages over regular garlic due to its higher amounts of organosulfide compounds in particular. This study aimed to determine the ability of SCGO extract to ameliorate hepatic steatosis and improve oxidative status by modulating expression of tumour necrosis factor α and superoxide dismutase in mice fed a HFD.

**Methods:**

Twenty-four adult male Balb/C mice were divided into six groups: i) normal diet; ii) positive control diet; iii) negative control diet; and iv) HFD with SCGO at 12.5 mg/kg body weight (mg/kg BW); v) HFD with SCGO at 25 mg/kg BW, vi) HFD with SCGO at 50 mg/kg BW. Liver weight and morphology, spleen weight, serum levels of superoxide dismutase (SOD) and tumour necrosis factor α (TNF-α), TNF-α expression in the aorta and lipid profiles were assessed at the end of the experimental period.

**Results:**

SCGO treatment was associated with significant decreases in liver and spleen weight as well as amelioration of hepatic steatosis. SCGO treatment also decreased TNF-α levels and expression. Serum levels of SOD in the SCGO groups were significantly increased compared with the negative control group. Lipid profiles were improved in the SCGO treatment groups compared with the negative control group.

**Conclusion:**

SCGO as an herbal medicine could be an effective treatment for degenerative disorders caused by HFD.

## Introduction

Consumption of a high-fat diet (HFD) causes increased lipid accumulation and contributes to various metabolic diseases such as obesity, hyperlipidemia, atherosclerosis, cardiovascular disease, type 2 diabetes mellitus and nonalcoholic fatty liver disease (NAFLD) (1–4). HFD contains lipids that are metabolised into triglycerides and cholesterol through exogenous and endogenous pathways in the intestine and liver, respectively (5, 6). HFD promotes metabolism via exogenous pathways that results in elevated levels of low-density lipoprotein (LDL) that can be converted to oxidised LDL (oxLDL). oxLDL interactions with blood vessel walls cause endothelium dysfunction (7, 8). Moreover, oxLDL stimulates formation of lipid peroxide free radicals, which, together with other free radicals, can cause a decrease in activity of antioxidant enzymes such as superoxide dismutase (SOD) (1, 9). Innate and adaptive immune system responses can also be activated in response to signaling mediated by oxLDL (10, 11). Increased levels of oxLDL promote differentiation of mononuclear cells (monocytes) into macrophages (8). Macrophages and dendritic cells that express Toll-like receptors that bind oxLDL scavenger receptors initiate an inflammatory signaling pathway by secreting pro-inflammatory cytokines such as tumour necrosis factor α (TNF-α) (12). The activation of inflammatory signaling pathways induces endothelial cells and smooth muscle cells to express adhesion molecules, chemoattractants, and growth factors and results in migration of leukocytes and smooth muscle cells in inflammatory blood vessel walls (10, 13).

Single clove garlic (SCG) is a tropical plant found in Indonesia that is frequently used as an herbal medicine. However, there are limited scientific reports concerning the health benefits of SCG. SCG has a strong alliaceous odour arising from the higher sulfide content than that of regular garlic (14). Naji et al. (15) reported that in rabbits with CCl_4_-induced hepatic damage, SCG had higher antioxidant capacity and elevated levels of 2,2-diphenyl-1-picrylhydrazyl (DPPH) to a greater degree than did regular garlic. SCG contains hydrophobic organosulfur compounds of S-allyl-L-cysteine sulfoxides (alliins). Alliins are converted into allicin by alliinase (16). Allicin is the precursor of various allyl sulfides such as diallyl disulfide (DADS), diallyl trisulfide (DATS), diallyl sulfide (DAS), E-ajoene, Z-ajoene, thioacroleins and vinyldithiins (14, 17). Single clove garlic oil (SCGO) extract is used as an herbal medicine that is thought to prevent and treat various diseases caused by HFD. Organosulfur in garlic can increase SOD activity and decrease secretion of pro-inflammatory cytokines such as TNF-α (17). The aims of this study were to determine whether SCGO extracts can ameliorate hepatic steatosis and improve oxidative status by decreasing levels of TNF-α and restoring superoxide dismutase levels in HFD.

## Methods

### SCGO Preparation

Single garlic was bought from the Ngadas local market in Malang, East Java, Indonesia. SCGO extracts were obtained using the soxhletation method (18). Briefly, 1 kg of single garlics was dried at 50 °C and then mashed to yield 0.5 kg of dried single garlic powder. The powder was placed in a thimble extractor with 2.5 L hexane as a solvent. The soxhletation process was carried out for 18 h and the resulting product was evaporated using a rotary evaporator for 3 h (19). This process yielded 3 mL SCGO extract that was used as a stock solution. The SCGO extract was diluted with corn oil to produce doses of 12.5 mg/kg BW, 25 mg/kg BW and 50 mg/kg body weight (BW) that were selected (based on previous research).

### Animal Model and Experimental Design

Male Balb/C mice were provided from CV Galaxy Science, Jember. To find the smallest informative sample population, the sample size was determined using Federer’s formula: *t*(*r*-1) > 15, where *t* and *r* are the number of treatments and replications, respectively (20). Based on these calculations, a total of 24 mice were used, with 4 mice in each group. The mice (25 ± 3 g, 10 ± 2 weeks old) were individually housed in standard cages and given free access to water and food. The animals were acclimatised for 7 days prior to beginning the experiment. After acclimatisation, the animals were randomly divided into normal diet and HFD and fed the respective diets for 45 days. The normal diet (PT, Comfeed, Indonesia) contained 60% carbohydrate, 16% protein, 3% fat, and 21% vitamins and minerals, while the HFD contained 8.84% carbohydrate, 8.5% protein and 34.2% fat, with the remainder consisting of fiber, vitamins and minerals. Simvastatin was purchased from DexaMedica (Tangerang, Indonesia).

At the end of the 45 days diet acclimatisation period, the animals were randomly divided into six groups with 4 mice each:

N: normal diet group
K−: HFD without treatment (negative control)
K+: HFD treated with 26 mg/kg BW statin (positive control)
P1: HFD treated with SCGO 12.5 mg/kg BW
P2: HFD treated with SCGO 25 mg/kg BW
P3: HFD treated with SCGO 50 mg/kg BW

**Figure 1:**
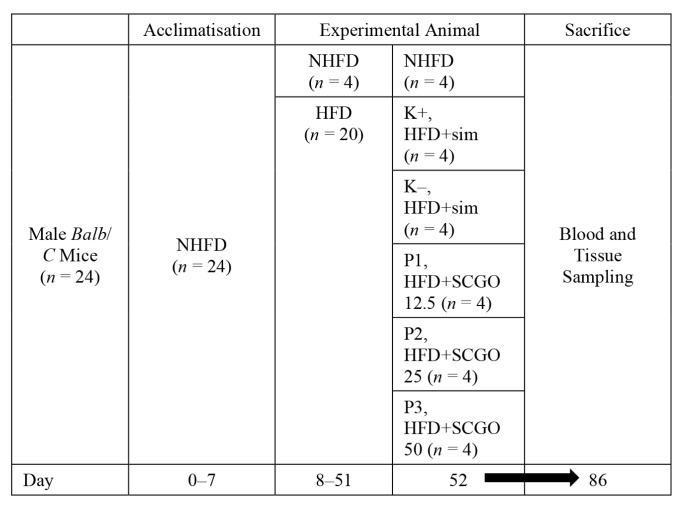
Experimental design

The indicated treatments were administered orally for 35 days. The experimental design of this study is shown in Figure 1. Animal welfare and experimental procedures were performed according to the Principles of Laboratory Animal Care and approved by the Brawijaya University Institutional Ethics Committee (approval No. 880-KEP-UB).

### Collection of Samples

After treatment, the animals were euthanised with 4% isoflurane. Blood collected by cardiac puncture was centrifuged and the resulting serum was stored at −20 °C prior to analysis. The liver and spleen were removed, weighed using an analytical balance and the tissue was fixed with 4% phosphate buffer saline paraformaldehyde (PBS-PFA).

### Histological Analysis

Liver tissues were fixed with 10% PBS-PFA, dehydrated and embedded in paraffin. The embedded tissue was cut into 5 μm-thick sections that were stained with hematoxylin-eosin (HE). Liver sections were observed under a light microscope (CX23, Olympus, Japan). Hepatic steatosis was assessed using the Nonalcoholic Steatohepatitis Clinical Research Network (NASH CRN) scoring system where 0–3.0: steatosis < 5%, 1: steatosis 5%–33%, 2: steatosis 34%–66% and 3: steatosis > 67% (21).

### Immunohistochemical (IHC) Examination

TNF-α expression in aorta tissue section slides was examined using IHC staining. Rat anti-TNF-α primary antibody (Cat. No. SC-52749, Santa Cruz Biotechnology, USA) was dissolved 1:1,000 in 2% bovine serum albumin (BSA) and incubated for 1 h. The secondary antibody was goat anti-rat immunoglobulin G (IgG) with fluorescein isothiocyanate (FITC) (Cat. No. 02-16-06, KPL, USA) that was dissolved in 2% BSA (1:1,500) and incubated for 1 h. The sections were washed three times with PBS for 8 min each. The slides were dried and mounted using Canada balsam and observed using a fluorescence microscope (FSX 100, Olympus, Japan). TNF-α expression is presented as intensity/mm^2^.

### Measurement of TNF-α Levels

An ELISA was used to measure TNF-α levels. In brief, 96-well plates were coated with 20 μL antigen serum produced by mixing serum with buffer coating (1:20) and incubating at 4 °C overnight. The plates were then washed with PBS + 0.2% Tween and incubated with primary antibody anti-TNF-α (Cat. No. SC-52749, Santa Cruz Biotechnology, USA) in the assay buffer (BSA+PBS) (1:1,000) for 2 h at room temperature before washing with PBS-0.2% Tween and coating with the secondary antibody anti-rat anti-TNF-α IgG biotin in assay buffer (1:2,000). The plates were incubated with the secondary antibody for 1 h at room temperature, washed with PBS-0.2% Tween and Streptavidin Horseradish (SA-HRP) enzyme in assay buffer (1:2,000) was added and incubated for 1 h at room temperature. The plates were then washed with PBS-0.2% Tween and SureBlue Toluene Methyl Benzidine (TMB) was added and incubated for 20 min at room temperature before 1N HCl stop solution was added. Absorbance at 450 nm was measured with an ELISA Reader (Bio-Rad Benchmark, Japan).

### Measurement of SOD Level

Serum SOD levels were measured using an ELISA kit for mouse SOD (Cat. No. E0290Mo, Bioassays Technology Laboratory, Shanghai, China) according to the manufacturer’s instructions.

### Statistical Analysis

Data for liver and spleen weight, TNF-α level and expression, and SOD level were analysed using one-way analysis of variance (ANOVA). The significance of differences between groups was calculated using the Duncan multilevel range test (DMRT) as a post-hoc test. Normal and homogeneous distribution were assumed. The data are presented as mean ± standard deviation of the means. *P*-value < 0.05 was considered significant.

## Results

### Effect of SCGO on Liver and Spleen Weight

The negative control group fed a HFD with no additional treatments showed significant increases in liver weight compared to the normal group fed a regular diet (*P* < 0.05). Meanwhile, SCGO treatments for 35 days resulted in a significant decrease in liver weight compared to both the negative control group (*P* < 0.05) and the statin-treated positive control group fed a HFD (Table 1).

Spleen weight was also significantly higher in the negative control group compared to the normal group. Mice fed a HFD supplemented with SCGO showed significantly decreased spleen weight compared to the negative control group (*P* < 0.05). However, the spleen weights for the SCGO treatment groups were not significantly different from mice in the normal and statin-treated groups (Table 1).

### Effect of SCGO on Hepatic Steatosis as Assessed by Histopathology

**Figure 2:**
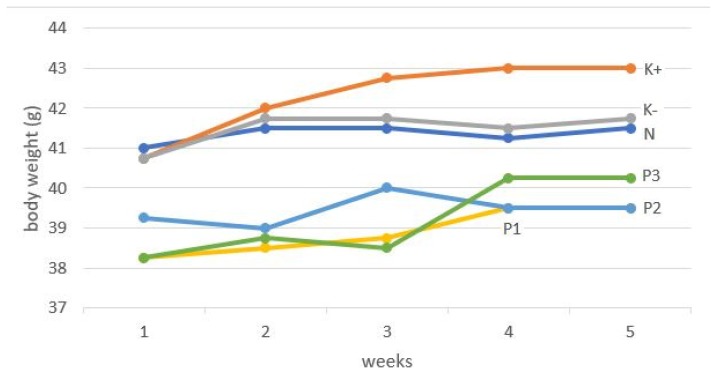
Body weight gain

**Figure 3:**
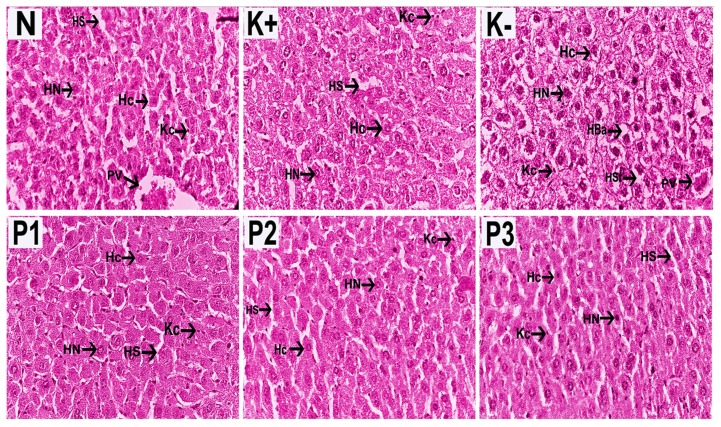
Histology of liver using H & E staining on normal and experimental groups (400× in magnification). Notes: Hc = hepatocyte; HN = hepatocytes nuclei; HS = hepatic sinusoids; KC = Kupffer cells; PV = portal vein; HBa = hepatic ballooning; HSt = hepatic steatosis

Liver sections were stained with HE to observe hepatic steatosis (fatty liver) characterised by the appearance of lipid droplets in hepatocytes. Liver sections from mice fed a normal diet had normal architecture without hepatic steatosis as opposed to those from the negative control group that showed large numbers of lipid droplets in liver cells, indicative of hepatic steatosis. Related with hepatic steatosis, different diet has an impact in body weight gain (Figure 2). Sections of livers from SCGO-treated mice showed ameliorated hepatic steatosis and hepatocytes that had a similar appearance to that seen in sections from normal mice (Figure 3).

### SCGO Treatment Decreases TNF-α Level in Serum and TNF-α Expression in Aorta

**Figure 4:**
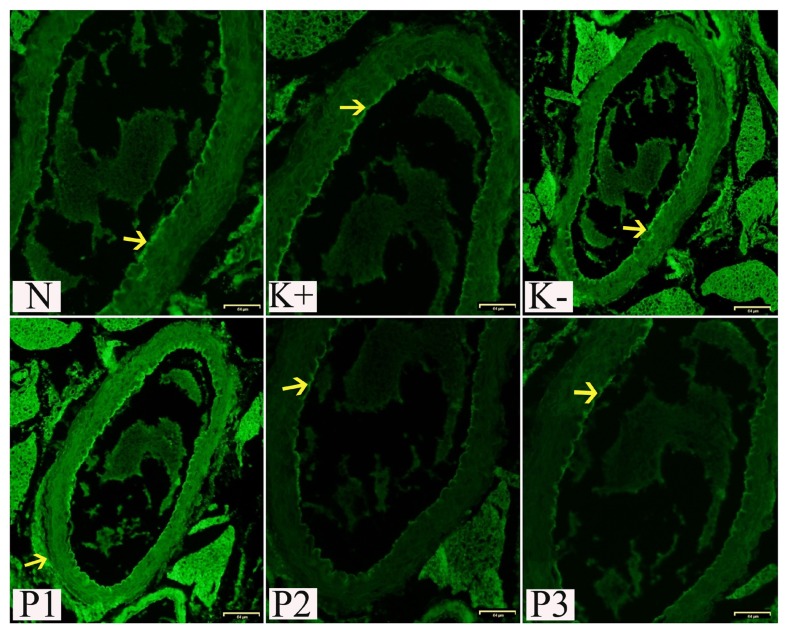
IHC of TNF-α expression in aorta on normal and experimental groups

TNF-α levels in serum were significantly increased in the negative control group compared to the normal group (*P* < 0.05). Treatment with SCGO was associated with a significant decrease in TNF-α levels compared to the negative control group. IHC indicated that mice treated with 12.5 mg/kg BW SCGO had high expression of TNF-α in the aorta, although these levels were not significantly different from those seen for mice fed HFD without SGCO. Meanwhile, treatment with 25 mg/kg BW and 50 mg/kg BW of SCGO did significantly decrease the TNF-α expression in the aorta (Table 1 and Figure 4).

### Increases in SOD Levels with SCGO Treatment

The serum SOD levels in the negative control group were significantly decreased compared to the normal group (*P* < 0.05). Mice fed a HFD and treated with SCGO had significant increases in serum SOD levels compared to the negative control group (Table 1).

### Effect of SCGO on Lipid Profiles

Lipid profiles including total cholesterol, LDL, triglyceride and HDL level in blood serum were measured (Table 1). The total cholesterol, LDL and triglyceride level in the negative control group were the highest among the groups, whereas the HDL level was the lowest for the negative control group. SCGO treatment significantly decreased levels of total cholesterol, LDL and triglyceride in the blood serum and increased the amount of unsaturated lipids, as reflected by increased HDL levels.

## Discussion

Metabolism of HFD either in exogenous or endogenous pathways can increase the level of fatty acid in blood circulation and promote elevations in triglycerides that are carried by VLDL to adipose tissue. Such features, including higher levels of LDL, total cholesterol and triglyceride were seen here for the negative control group fed a HFD. On the other hand, increased adipose tissue volume can also increase body weight gain. A study by Cho et al. (22) reported that the HFD experimental group had the highest level of LDL, total cholesterol and triglyceride, but the lowest levels of HDL. Consumption of HFD causes increased fatty acid synthesis in the liver that results in lipid accumulation that disrupts metabolic homeostasis in the liver. Lipid accumulation causes cellular damage in the liver that causes inflammation, necrosis, fibrosis and liver cirrhosis (3, 6). In the present study, the liver weight of the negative control group fed a HFD was significantly higher compared to that for the normal group. HE staining of hepatic liver tissue also showed that the negative control group had higher numbers of lipid droplets than did the normal group. These results are consistent with those of a study by Meli et al. (23) that revealed progressive increases of hepatic steatosis and inflammatory damage in the liver of rats fed a HFD. Zheng et al. (24) also reported that mice fed a HFD has higher steatosis scores, inflammation, ballooning, and NAFLD in the liver.

Accumulation of lipids caused by HFD consumption and hepatocyte damage (steatosis) can trigger innate and adaptive immune responses (25). Increasing the amount of lipids, particularly that of oxLDL, in the blood affects spleen function by promoting maturation and proliferation of macrophages, B cells and T cells induced by inflammatory signaling processes (2, 25–27). The results of this study showed that the negative control group had a higher average spleen weight than the normal group. Gomaa and El-Aziz (25) reported that splenic sections from rats fed a HFD had white pulp containing lymphocytes around a central arteriole. B and T cell activation as well as accumulation of B memory cells all occur in the white pulp region in the spleen. Erythrocytes and macrophages also formed splenic cords that contributed to increased spleen weight. Furthermore, Torello et al. (28) reported that obese mice fed a HFD had significantly increased numbers of granulocyte-macrophage progenitors (CFU-GM) in the spleen compared to the control group.

oxLDL can trigger dendritic cells and macrophages to express various receptors such as scavenger receptors (SRs), toll-like receptors (TLRs) and nucleotide-binding oligomerisation domains (NOD)-like receptors (NLRs) that recognise oxLDL. TLR4 expressed on dendritic cells or macrophages binds LDL receptors (LDLr) to activate an inflammatory signaling pathway by promoting secretion of pro-inflammatory cytokines such as TNF-α (11, 29). Here we found that the serum level of TNF-α and TNF-α expression in the aorta in the negative control group fed a HFD was higher than that in the normal group. Jovinge et al. (30) observed in a mouse model of atherosclerosis that increased activation and TNF-α secretion by macrophages was associated with oxLDL, whereas native LDL did not stimulate TNF-α release.

Here we found that the negative control group fed a HFD had lower SOD levels than the normal group, which is consistent with findings by Yu et al. (31) that rats fed a hyperlipidemic diet had decreased levels of SOD in the serum and liver. HFD consumption increases the amount of cholesterol and LDL in blood circulation. This increased LDL may in turn mediate activation of reactive oxygen species (ROS) and nicotinamide adenine dinucleotide phosphate (NADPH) oxidase in mitochondria. ROS are free radicals that can promote formation of other radicals such as lipid peroxide. Increased amounts of free radicals can decrease activity of endogenous antioxidants such as SOD that can prevent free radical-induced damage (1, 9, 32).

Treatment with SCGO increased SOD levels compared to the negative control group. SCGO contains large amounts of various oil-soluble organosulfurs such as allicin, alliin and ajoene. Allicin is an active compound that can increase endogenous antioxidant activity of SOD (16, 33). Increasing the activity of antioxidant enzymes like SOD can decrease levels of lipid peroxides acting as free radicals. SOD can also convert the free radical anion superoxide (2O_2_^•−^) into H_2_O_2_ whereas catalase promotes conversion of H_2_O_2_ into H_2_O and O_2_ (11, 32, 34, 35). Meanwhile, Mohebbi et al. (36) reported that increased SOD activity was followed by an increase in glutathione peroxidase (GSPHx) enzyme activity.

Binding of TNF-α to TNFR1 can trigger inflammatory signaling pathways. This binding activates the transcription factors nuclear factor kappa B (NF-κB) and c-Jun/ATF-2 to enhance recruitment of leukocytes including monocytes, B cells and T cells (13, 37). NF-κB is a transcription factor involved in synthesis of the pro-inflammatory cytokine TNF-α. Allicin in SCGO extracts has been shown to inhibit NF-κB activation that in turn decreases TNF-α levels. Allicin can promote increases in SOD levels that downregulate oxLDL-mediated effects on dendritic cells and macrophage recognition receptors such as TLR4. Down-regulation of TLR4 can decrease production of TNF-α. SCGO also contains ajoene that has anti-thrombosis and anti-aggregation activities by preventing binding of platelets to fibrinogen receptors that in turn prevents formation of thrombi in atherosclerosis disease (17). Here we showed that treatment of mice fed a HFD with SCGO could decrease TNF-α levels in serum compared to the negative control group given HFD alone. SCGO treatment at 25 mg/kg BW and 50 mg/kg BW was associated with decreased TNF-α expression in the aorta compared to the negative control group. However, treatment with SCGO 12.5 mg/kgBW did not significantly decrease TNF-α expression in the aorta relative to the negative control group. Gao et al. (33) reported that organosulfur compounds in garlic such as allicin, alliin, and ajoene were highly distributed in serum, liver and kidney. High expression levels of TNF-α in the aorta are likely caused by the distribution of organosulfur compounds in the aorta such that higher doses would be needed to decrease TNF-α expression in this tissue.

## Conclusion

SCGO at 25 mg/kg BW and 50 mg/kg BW given to mice fed a HFD was a potential alternative treatment to ameliorate oxidative status by increasing the activity of SOD antioxidant enzymes and decreasing levels of pro-inflammatory cytokines such as TNF-α that are caused by a HFD. The improvements in metabolism were manifested as decreases in liver and spleen weight and amelioration of hepatic steatosis.

## Figures and Tables

**Table 1:** Biochemical profiles, organ weights and lipid profile of experimental group

	Experimental Groups						*P*-value
	*N* (*n* = 4)	K+ (*n* = 4)	K− (*n* = 4)	P1 (*n* = 4)	P2 (*n* = 4)	P3 (*n* = 4)	
Liver weight (g)	1.32^a^ (0.09)	2.14^cd^ (0.18)	2.26^d^ (0.19)	1.83^b^ (0.07)	1,72^bc^ (0,04)	1,88^bc^ (0,07)	0.001
Spleen weight (g)	0.21^a^ (0.02)	0.23^a^ (0.04)	0.35^b^ (0.03)	0.21^a^ (0.02)	0.18^a^ (0.01)	0.19^a^ (0.03)	0.004
SOD level (ng/mL)	24.88^c^ (0.55)	22.73^b^ (0.60)	20.15^a^ (1.24)	25.26^c^ (0,31)	26.52^cd^ (0,83)	28.27^d^ (0.34)	0.000
TNF-α level (pg/mL)	478.75^a^ (106.74)	641.25^a^ (112.44)	1272.50^b^ (204.51)	585.00^a^ (47.87)	597.50^a^ (96.55)	710.00^a^ (102.06)	0.003
TNF-α expression (int/mm^2^)	47.62^c^ (3.25)	42.09^b^ (1.60)	54.71^d^ (1.00)	59.80^d^ (0.89)	34.79^a^ (1.36)	32.10^a^ (0.95)	0.000
Total cholesterol (mg/dL)	122.33^ab^ (27.79)	99.33^a^ (8.14)	266.33^c^ (26.38)	118.67^ab^ (14.57)	127.00^ab^ (23.64)	156.33^b^ (20.23)	0.000
LDL (mg/dL)	76.00^ab^ (14.73)	62.33^a^ (16.65)	166.67^c^ (12.66)	88.00^b^ (16.64)	80.00^ab^ (4.00)	89.33^b^ (7.09)	0.000
Triglyceride (mg/dL)	82.33^a^ (4.04)	75.67^a^ (13.31)	156.33^b^ (17.09)	94.00^a^ (7.55)	75.00^a^ (13.00)	93.00^a^ (6.24)	0.000
HDL (mg/dL)	54.60^b^ (7.20)	62.67^bc^ (5.51)	39.17^a^ (4.25)	58.97^b^ (2.70)	66.67^c^ (6.11)	66.00^c^ (5.00)	0.000

Notes: The different letters (a, b, c and d) indicate significance compared to another (*P* < 0.05)
